# Ultrafast Thermal RAFT Depolymerization at Higher Solid Contents

**DOI:** 10.1021/acsmacrolett.5c00009

**Published:** 2025-02-10

**Authors:** Dimitra Mantzara, Richard Whitfield, Hyun Suk Wang, Nghia P. Truong, Athina Anastasaki

**Affiliations:** Laboratory of Polymeric Materials, Department of Materials, ETH Zurich, Zurich, 8093, Switzerland

## Abstract

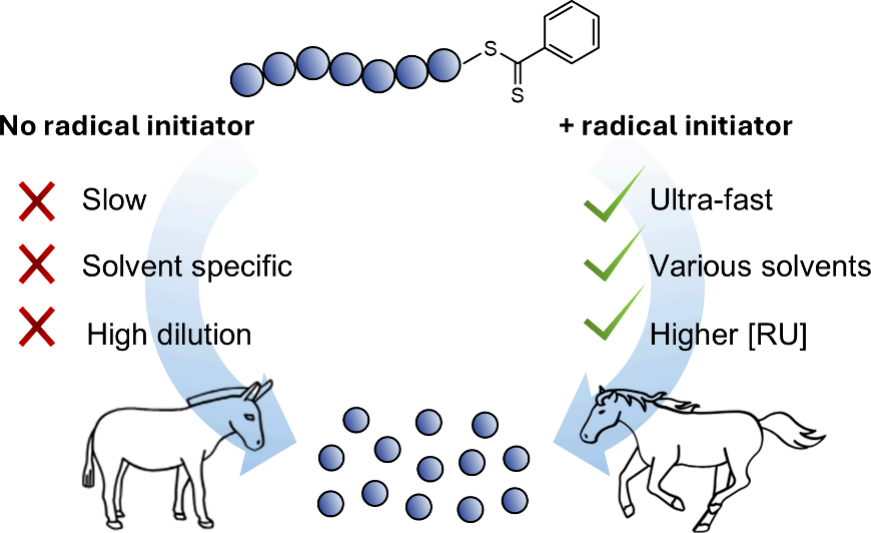

Although thermal solution RAFT depolymerization has recently emerged as an efficient chemical recycling methodology, current approaches require specialized solvents (i.e., dioxane), typically suffer from extended reaction times, and operate exclusively under highly dilute conditions (i.e., 5 mM repeat unit concentration). To circumvent these limitations, a commercial radical initiator is introduced to kinetically untrap the depolymerization and promote chain-end activation. By varying the initiator concentration, a remarkable rate acceleration (up to 72 times faster) can be observed, enabling the completion of the depolymerization within 5 min. Notably, a 20-fold increase in the repeat unit concentration did not appreciably compromise the final depolymerization yield, while very high percentages of monomer could be recovered in a wide range of solvents, including dimethyl sulfoxide, anisole, xylene, acetonitrile, toluene, and trichlorobenzene. Our findings not only offer intriguing mechanistic aspects, but also significantly expand the scope and applications of thermal RAFT depolymerization.

The labile end-groups installed by controlled radical polymerization (CRP), also referred to as reversible deactivation radical polymerization (RDRP),^1−7^ have recently been leveraged to promote efficient depolymerizations at temperatures lower than those typically required for materials prepared by conventional radical polymerization (<170 °C vs >350 °C).^8−17^ Activation of the chain-end generates a radical that, under thermodynamically favorable conditions, can trigger the rapid depropagation of the polymer chain.^10^ Initial pioneering work by the groups of Raus and Gramlich^18,19^ demonstrated the feasibility of chain-end depolymerizations during the atom transfer radical polymerization (ATRP) and reversible addition–fragmentation chain-transfer (RAFT) polymerization of bulky monomers, highlighting that competing depolymerization equilibria may limit the overall monomer conversion. This work was further supported by Matyjaszewski and co-workers who reported the copper-catalyzed depolymerization of a Cl-capped poly(poly(dimethylsiloxane) methacrylate).^20^ More recently, the depolymerization of less specialized and nonbulky polymers has attracted considerable attention.^5,14,21−28^ Ouchi and co-workers were the first to show the depolymerization of poly(methyl methacrylate), resulting in up to 24% of monomer recovery in the presence of a ruthenium catalyst.^9,29^ Significantly enhanced yields (i.e., 67–76%) were subsequently obtained by Matyjaszewski’s group at 170 °C when either copper or iron catalytic systems were employed.^30,31^ In 2023, our group in collaboration with the Matyjaszewski group introduced the first photothermal ATRP depolymerization methodology whereby 90% of monomer was successfully recovered during the depolymerization of poly(benzyl methacrylate).^32^

A particularly noteworthy aspect of ATRP depolymerizations is that they can proceed effectively even at relatively high concentrations (i.e., 750 mM repeat unit (RU) concentration), while reaching completion in less than 10 min.^13,32,33^ Instead, RAFT depolymerizations require prolonged reaction times and are nearly exclusively performed under more dilute conditions (i.e., [RU]_0_ = 5 mM). For example, our group reported the first efficient depolymerization of PMMA at 120 °C, reaching very high monomer conversions (86%), albeit at [RU]_0_ = 5 mM.^34−39^ The reaction required over 4 h to reach near-quantitative levels, and significantly lower yields (i.e., 10%) were obtained at higher loadings (i.e., [RU]_0_ = 250 mM), thus highlighting the sensitivity of the system to the polymer concentration.^34,35^ To accelerate the reaction, Sumerlin and co-workers and our group independently utilized light as an external stimulus, although very low RU concentrations (i.e., 5 mM) were also necessary to achieve high conversions.^40,41^ It is worth noting that the overwhelming majority of RAFT depolymerizations necessitate the use of specialized solvents, capable of providing solvent-derived radicals that can trigger an efficient depolymerization (i.e., dioxane), while minimal conversions have been reported in other solvents, thus significantly limiting the applicability and scope of these depolymerizations.^35−38,41^

Considering that both ATRP and RAFT methodologies focus on identical methacrylic-based materials, and that the equilibrium monomer concentration is independent of the polymer end-group,^10,42^ we hypothesized that a kinetic barrier in the activation step was preventing the RAFT depolymerization from reaching comparable yields in shorter timeframes, and at higher polymer loadings. To test this hypothesis, we report here the use of a radical initiator to promote chain-end activation, resulting in an ultrafast RAFT depolymerization that can effectively proceed at higher polymer concentrations and in a wide range of solvents (Scheme 1).

**Scheme 1:**
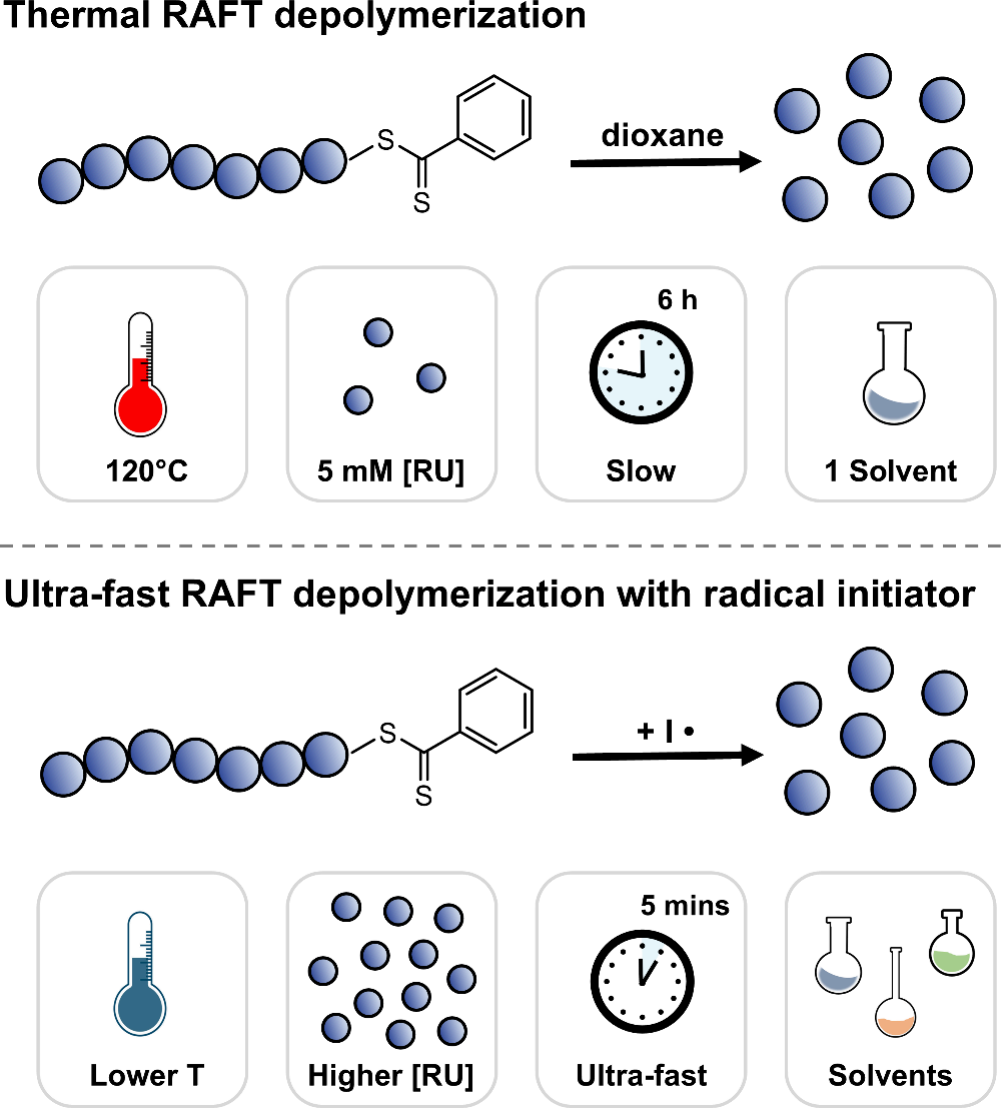
Comparison between Previous Approaches and the Current Approach to Depolymerize Polymers Synthesized by RAFT Polymerization

Poly(benzyl methacrylate) (PBzMA) was initially synthesized via conventional RAFT polymerization in acetonitrile at 70 °C, using a dithiobenzoate as the RAFT agent and azobis(isobutyronitrile) (AIBN) as a radical initiator (Scheme S1).^4,43,44^ The resulting polymer exhibited excellent control over the molar mass distribution (*Đ* = 1.19), an overall livingness of 98% (eq S1), and served as our model polymer for this study upon rigorous purification (Figures S1–S3). 1,2,4-Trichlorobenzene (TCB) was chosen as a representative poorly performing solvent as the thermal RAFT depolymerization of PBzMA at [RU]_0_ = 5 mM, resulted in a rather slow depolymerization at 120 °C (Scheme S2, Figure 1a, gray). Specifically, within 10 min, only 11% (Table S1) of monomer was recovered. After 2 h, the percentage of regenerated monomer increased to 23% but the conversion did not surpass 28%, even after 8 h of reaction time as measured by proton nuclear magnetic resonance (^1^H NMR) spectroscopy (Table S1, Figure S4). The experiment was then repeated in the presence of 0.2 equiv of azobis(cyclohexanecarbonitrile) (ABCN), and under otherwise identical conditions (Scheme S3). Detailed kinetic analysis revealed a remarkable rate acceleration with 73% of monomer obtained in the first 10 min, while the reaction reached 87% after approximately 30 min. This corresponded to a 46-fold rate increase (Tables S2 and S3, Entry 1, and Figure 1a,b, blue). No further depolymerization could be detected after 1 h, while the UV-SEC detector confirmed the disappearance of the UV signal, thus indicating that the depolymerization had reached completion (Table S3, Entry 1, Figure S5). Encouraged by these exciting data, the effect of various ABCN concentrations on the depolymerization outcome was investigated (Table S3, Entries 2–5, Figure S6). While 0.5 and 1 equiv of ABCN further increased the reaction rate (72 times higher rate compared to reaction with no initiator), without compromising the final monomer yield (>80% within 10 min), when even higher amounts of ABCN were employed (i.e., 2 and 5 equiv), a lower percentage of monomer was obtained (as low as 68%, Table S3, Figures 1b and S6). This lower percentage of recovered monomers is attributed to increased side reactions and termination events imposed by the excess of radicals introduced into the system. Nevertheless, our preliminary data unambiguously demonstrate that the addition of a judiciously optimized small amount of radical initiator not only enhanced the depolymerization rate but also boosted the final depolymerization conversion. These advantageous characteristics were attributed to enhanced chain-end activation and subsequent fragmentation, promoted by the radical initiator which provided a rapid and steady radical supply (Scheme S4). This steady supply of radicals omits the use of a specialized solvent as a radical provider and improves reproducibility potentially caused from batch-to-batch dioxane variations.^36^

**Figure 1:**
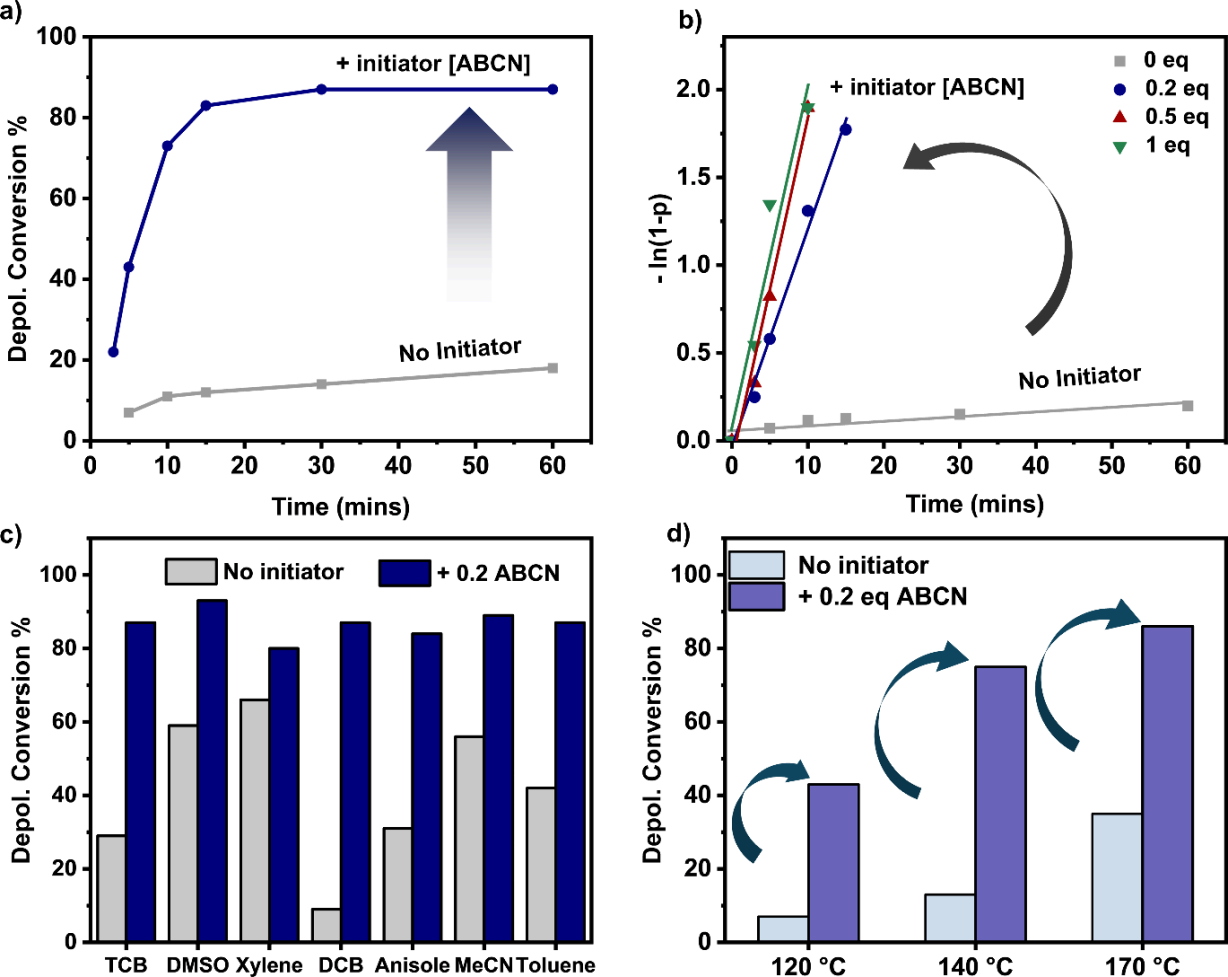
Effect of the radical initiator on RAFT depolymerization. Presented are (a) a comparison between the depolymerization kinetics in the absence and presence of 0.2 equiv of ABCN, (b) the effect of the amount of ABCN on the rate of the depolymerization, (c) the effect of the addition of ABCN on depolymerization in various solvents after 2 h of reaction time, and (d) the effect of the temperature on the depolymerization conversion after 5 min of reaction time. All reactions were performed at [RU]_0_ = 5 mM.

To investigate the potential of our approach to operate in various reaction media, a series of solvents were sequentially employed, namely, 1,2-dichlorobenzene (DCB), xylene, acetonitrile, anisole, dimethyl sulfoxide (DMSO) and acetonitrile (MeCN, Figure 1c). In all cases, very high monomer regeneration (>80%) was achieved in 2 h in the presence of 0.2 equiv of ABCN (Table S4, Figure 1c, blue). Instead, the control experiments without a radical initiator demonstrated notably lower conversions (Table S5, Figure 1c, gray). For example, the thermal depolymerization of PBzMA at 120 °C in DCB resulted in only 9% of conversion after a comparable time frame, thus highlighting the superiority of our methodology. Notably, the rate of depolymerization could also be increased in dioxane in the presence of the initiator. In the absence of radical initiator, only 20% of conversion was detected in 15 min, while the addition of 0.2 equiv of ABCN led to 86% monomer regeneration within the same time frame (Table S6 and Figure S7). It is worth highlighting that the very high conversions achieved within a short time scale are comparable with those obtained by thermal ATRP depolymerizations; albeit the later ones have almost exclusively been performed at much higher temperatures (170 °C).^31,33^

To aid a fairer comparison, a series of thermal RAFT depolymerizations were conducted at 140 and 170 °C, while keeping the radical initiator concentration constant (i.e., 0.2 equiv, Figure 1d). As expected, even higher reaction rates were obtained at these higher temperatures, resulting in near quantitative yields at 10 and 5 min, respectively (>85% depolymerization conversion, Tables S7–S9 and Figures S8 and S9). These results compare favorably with the control experiments without a radical initiator (Table S9, Figure S9). At 140 °C and in the absence of ABCN, only 13% of monomer could be detected in 5 min, with a maximum conversion of 50% obtained in 2 h (Table S10, Entry 1). Similar results were obtained at 170 °C, highlighting compromised rates and yields in the absence of a radical initiator (Table S10, Entry 2). It is noted that the addition of a radical initiator was also advantageous at temperatures lower than 120 °C. For example, at 100 °C, 81% of monomer could be successfully regenerated after 3 h (Table S11, Figure S10), while at 80 °C, with AIBN as the initiator, the conversion was 58%, indicating that significant monomer regeneration could be achieved even at this low temperature (Table S12, Figure S11). Taken altogether, our results demonstrate that by introducing a small amount of a radical initiator, higher rates of depolymerization were recorded and the final yield could be maximized at various reaction temperatures.

Considering the enhanced rates and yields at [RU]_0_ = 5 mM, we subsequently hypothesized that much higher percentages of recovered monomer may also be obtainable at higher polymer loadings. First, a series of thermal RAFT depolymerizations were conducted without a radical initiator and at various higher RU concentrations (Figure 2a). When the depolymerization was performed at 120 °C with a [RU]_0_ = 10 mM, only 17% of monomer was recovered in 2 h and this percentage decreased further to 15%, 14%, and 12% when [RU]_0_ of 25, 50, and 100 mM were employed, respectively (Table S13, Entry 1, Figure 2e, pale blue). At even higher concentrations (i.e., [RU]_0_ = 250 and 500 mM) negligible, if any, depolymerization was achieved (<7%), as shown in Figure 2e, light blue (Table S13, Entry 1). Notably, the introduction of 0.2 equiv of ABCN had a remarkable effect on the reaction’s yield with 72% of recovered monomer at [RU]_0_ = 100 mM (Table S13, Entry 2, Figure 2b, dark blue). This is a 6-fold increase in the final depolymerization yield, thus demonstrating the robustness of the system to operate at higher polymer concentrations efficiently. Even at the highest studied RU concentrations of 250 and 500 mM, 50% and 35% of retrieved monomer could be achieved (Table S13, Entry 2, Figure 2e). It is noted that previous thermal RAFT depolymerization strategies yielded only 10% of monomer at [RU]_0_ = 250 mM, thus highlighting the superiority of this methodology to operate at higher polymer loadings.^34,35^ The temperature was then increased from 120 to 140 °C, whereby the addition of ABCN resulted in >80% of depolymerization at [RU]_0_ = 100 mM, as opposed to only 18% conversion when the radical initiator was not employed (Table S14, Figure 2c,f). This successful trend was also reflected at higher RU concentrations, whereby at [RU]_0_ = 250 and 500 mM, final yields of up to 68% and 54% were possible, as opposed to the control experiments that resulted in only 13% and 11%, respectively (Table S14, Entry 1, Figure 2f). Finally, thermal RAFT depolymerizations were also performed at 170 °C. Despite the much higher reaction temperature, omission of the radical initiator yielded just 41%, 30%, and 23% conversion at [RU]_0_ = 100, 250, and 500 mM, respectively (Table S15, Entry 1, Figure 2d,g). This suggests that the rate of solvent-derived radical generation, even at this significantly higher temperature, is insufficient to trigger an efficient depolymerization. Instead, the addition of ABCN resulted in increased final conversions of 82%, 78%, and 61% from [RU]_0_ = 100 to 500 mM, respectively (Table S15, Entry 2, Figure 2g). As such, it can be universally concluded that irrespective of the temperature and repeat unit concentration employed, enhanced conversions and yields can be obtained in all cases.

**Figure 2:**
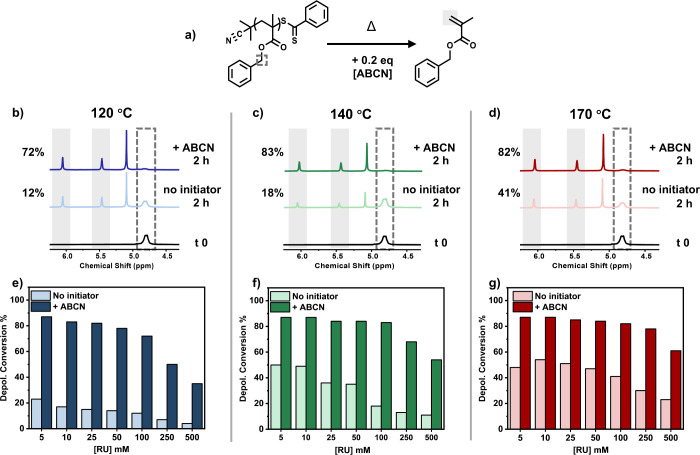
Effect of the initiator on RAFT depolymerization at various repeat unit concentrations (5–500 mM). In (a), a scheme of the reaction is illustrated. In (b)–(d), ^1^H NMR illustrate the final depolymerization conversions for reactions performed at [RU]_0_ = 100 mM, with and without 0.2 equiv of ABCN at 120 °C, 140 °C and 170 °C, respectively. In (e)–(g), final depolymerization conversions are presented for the various repeat unit concentrations and temperatures. All reactions were sampled after 2 h.

To summarize, in this work we have shown that by introducing a commercially available radical initiator such as ABCN, chain-end activation is being promoted, resulting in higher percentages of recovered monomer and faster rates of depolymerization. In particular, within 10 min the depolymerization can reach completion at temperatures equal to or higher than 120 °C, while significant monomer regeneration can also be obtained at lower temperatures. These rates are as much as 72 times faster when compared to the control experiments in the absence of a radical initiator. The versatility and robustness of this methodology were demonstrated by its compatibility with different solvents, all of which resulted in near-quantitative depolymerizations. Importantly, the presence of the radical initiator also pushed the reaction to proceed more effectively at higher polymer loadings with appreciable monomer recovery occurring even at [RU]_0_ = 500 mM, thus paving the way for many further opportunities.

## Supporting Information Available

Experimental section (PDF)
