# Supplementary material for: Ultrafast Thermal RAFT Depolymerization at Higher Solid Contents
Source: ACS Macro Lett. 2025 Feb 10;14(3):235–40. doi: 10.1021/acsmacrolett.5c00009 (PMC11924316; doi:10.1021/acsmacrolett.5c00009)
Supplement: Supplementary file 1 — mz5c00009_si_001.pdf [file mz5c00009_si_001.pdf]

Supporting information

Ultra-fast Thermal RAFT Depolymerization at Higher Solid Contents

Dimitra Mantzara, Richard Whitfield, Hyun Suk Wang, Nghia P. Truong, Athina Anastasaki*

Laboratory of Polymeric Materials, Department of Materials, ETH Zurich, Zurich, 8093, Switzerland.

Experimental

Materials:

Benzyl methacrylate (>98.0%) was purchased from Tokyo Chemical Industry. It was purified by filtration through basic alumina before use. The remaining materials were purchased either from Sigma-Aldrich or Fischer Scientific and were used as received.

Instrumentation:

Proton nuclear magnetic resonance spectroscopy:

^{1}H-NMR spectra were measured on a 500MHz Bruker Avance III HD spectrometer equipped with a BBO cryoprobe prodigy. Deuterated chloroform ($CDCl_3$) was used as the solvent. The depolymerization conversions were calculated by comparing the integrals of the monomeric vinyl proton (5.6 –6.2 ppm) peaks to the integrals of the combined monomer and polymer methylene peaks (4.9 –5.2 ppm).

Size exclusion chromatography (SEC)

Size exclusion chromatography (SEC) was conducted using a Shimadzu modular system, which includes a CBM-20A system controller, an SIL-20A automatic injector and a 10.0 µm bead size guard column (50 × 7.5 mm) connected to three KF-805L columns (300 × 8 mm, 10 µm bead size, with a maximum pore size of 5000 Å) and an RID-20A differential refractive index detector. N,N-Dimethylacetamide (HPLC grade, containing 0.03% w/v LiBr) was used as the mobile phase, with the flow rate maintained at 1 mL/min using an LC-20AD pump. The molecular weight calibration was performed using narrow molar mass distribution poly(methyl methacrylate) standards, with molecular weights ranging from 5000 to 1.5×10^6 Da (Agilent Technologies). All SEC samples were dissolved in dimethylacetamide and filtered through and a 0.45 µm PTFE filter prior to analysis.

General Procedures:

Synthesis of PBzMA-DTB polymer

In a 50 mL round bottom flask equipped with a magnetic stir bar, 326.6 mg of 2-cyanoprop-2-yldithiobenzoate (1.47 mmol, 1 equiv.) were added and dissolved in 7.0 mL of acetonitrile, resulting in a bright red solution. A stock solution (1 ml) of AIBN (48.4 mg) was prepared, out of which 0.5 mL (0.147 mmol, 0.1 equiv.) were transferred to the round bottom flask. Afterwards, 15 mL of benzyl methacrylate (88.5 mmol, 60 equiv.) was added, and the flask was sealed with a rubber septum. The polymerization mixture was deoxygenated via nitrogen purging for 20 minutes. The flask was later placed into a 70 °C oil bath for 2 hours and 40 minutes. Samples for ^{1}H-NMR and SEC analysis were taken periodically under nitrogen. Polymerization was stopped at 65% polymerization conversion by removing the reaction from the oil bath and removing the septum, exposing it to air.

Purification of PBzMA-DTB polymer

The polymer was precipitated in cold methanol and filtered under vacuum. This process was repeated 3 times, and the purified polymer was dried in the vacuum oven for 24 hours.

General depolymerization procedure for PBzMA-DTB without radical initiator

In a 21 mL test tube, 5.48 mg of PBzMA was added and then dissolved in 6 mL of 1,2,4-trichlorobenzene (5 mM BzMA repeat unit concentration). The test tube was sealed with a rubber septum and deoxygenated via nitrogen purging for 20 minutes. Subsequently, it was submerged in a 120 °C oil bath to start the depolymerization reaction. Samples for ^{1}H-NMR were taken periodically *in situ* under a nitrogen blanket.

General depolymerization procedure for PBzMA-DTB with radical initiator

In a 21 mL test tube 5.48mg of PBzMA (1 equiv.) was added and dissolved in 5.95 mL of 1,2,4-trichlorobenzene. A stock solution of ABCN was prepared (2 mg ABCN / 2.3 mL TCB), 0.05 mL (0.2 equiv.) of which was transferred to the reaction solution. The test tube was sealed with a rubber septum and deoxygenated via nitrogen purging for 20 minutes. Subsequently, the test tube was submerged in a 120 °C oil bath to start the depolymerization reaction. Samples for ^{1}H-NMR analysis were taken periodically under nitrogen blanket.

The same depolymerization procedure was repeated with different amounts of initiator, various solvents, various temperatures and different polymer loadings.

Livingness calculation

The theoretical livingness of the synthesized polymer was calculated to be 98% using the equation below.

$$L = \frac{[CTA]_0}{[CTA]_0 + 2f[\mathrm{I}]_0\,(1 - e^{-k_d \mathrm{t}})(1 - \frac{f_c}{2})}$$

Equation S1: Theoretical livingness equation[1]

Where L is the livingness, $[CTA]_0$ is the concentration of RAFT agent, f is the initiation efficiency, $[I]_0$ is the concentration of the initiator at time zero, k_d is the decomposition constant of the initiator, t is the reaction time, and f_c is the coupling factor. For the calculations we set: $[CTA]_0/[I]_0= 10$, $f= 0.5$, $k_d= 0.000032$, t= 9,600 s, $f_c= 0.25$.

Initiator decomposition profiles

ABCN 10 h half-life temperature: 88°C

ABCN 1 h half-life temperature: 105°C

AIBN 10 h half-life temperature: 65°C

AIBN 1 h half-life temperature: 82°C

Polymerization of BzMA

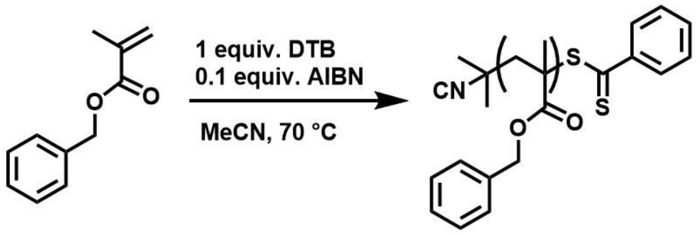

Scheme S1: RAFT polymerization of benzyl methacrylate with 2-cyano-2-propyl dithiobenzoate as the chain transfer agent. Reaction was performed with a [BzMA]:[CTA]:[AIBN]= 60:1:0.1 in acetonitrile (0.5:1 solvent to monomer volume ratio) at 70 °C.

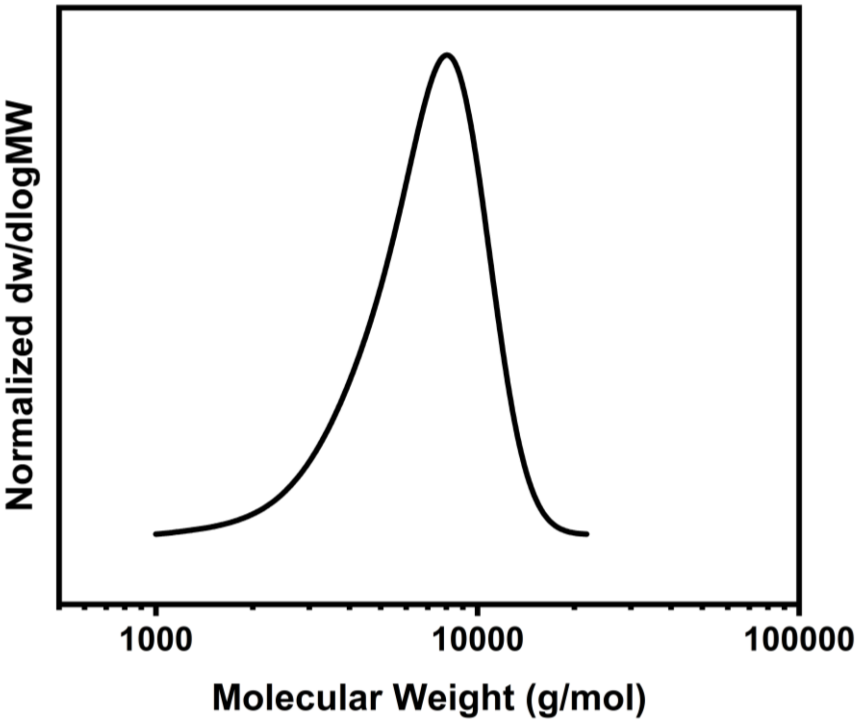

Figure S1: SEC trace of PBzMA-DTB synthesized via RAFT polymerization with 2-cyano-2-propyl dithiobenzoate as the chain transfer agent. Reaction was performed with [BzMA]:[CTA]:[AIBN]= 60:1:0.1 and the final PBzMA was obtained with an M_n= 6,200 and a $Đ$= 1.19.

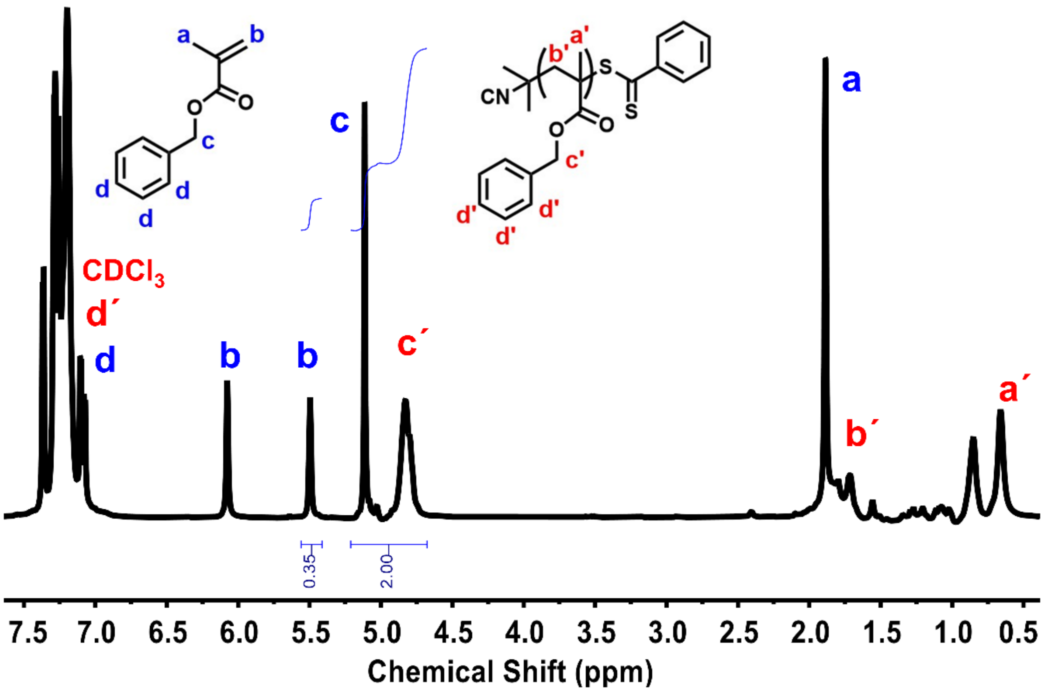

Figure S2: ^{1}H-NMR spectrum of the PBzMA-DTB polymer before purification. The polymerization conversion was calculated to be 65% after comparing the integrals of the monomeric vinyl proton (5.6 –6.2 ppm) peaks to the integrals of the combined monomer and polymer methylene peaks (4.9 –5.2 ppm).

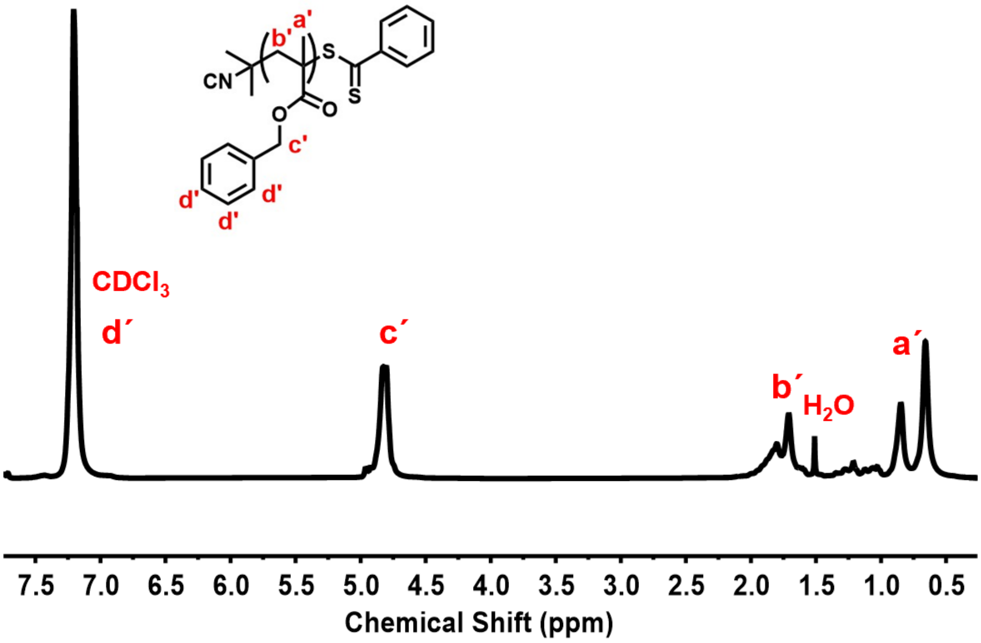

Figure S3: ^{1}H-NMR spectrum of purified PBzMA-DTB polymer.

Depolymerization of PBzMA-DTB

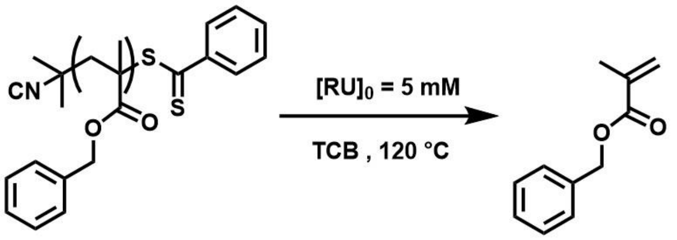

Scheme S2: Thermal RAFT depolymerization of PBzMA-DTB. Reaction was performed in TCB, at 120 °C with a $[RU]_0$= 5 mM.

Table S1: Kinetics of depolymerization of PBzMA in the absence of initiator. Reaction conditions were as follows: Solvent= TCB, T= 120°C, $[RU]_0$= 5 mM, V_{tot}= 6 mL. The first 60 minutes of data corresponds to what is presented in Figures 1a and b.

**Time (min)**	**Depol. (%) (NMR)**
**5**	7
**10**	11
**15**	12
**30**	14
**60**	18
**120**	23
**240**	26
**480**	28

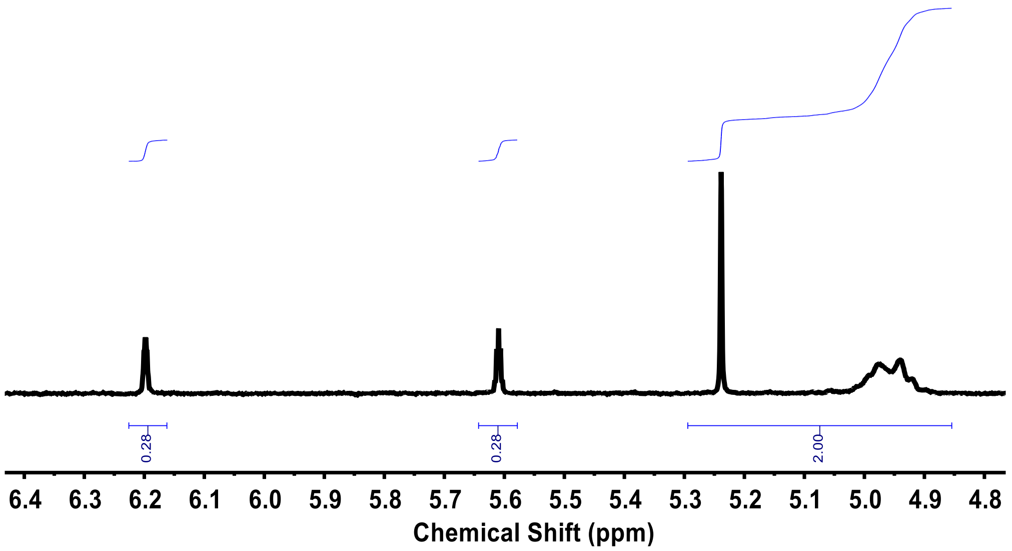

Figure S4: ^{1}H-NMR spectrum after 8h of the depolymerization reaction of PBzMA in TCB at 120 °C. Depolymerization conversion was calculated to be 28%. This was calculated by comparing the integrals of the vinyl monomer peaks (5.6 –6.2 ppm) to the integral of the combined monomer and polymer methylene peaks.

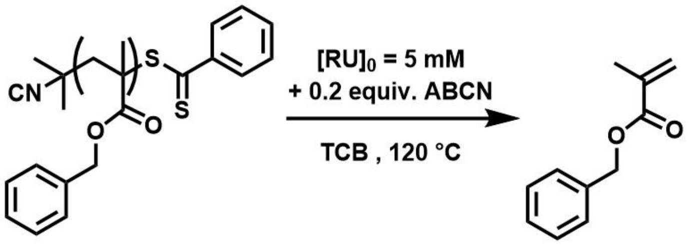

Scheme S3: Thermal RAFT depolymerization of PBzMA-DTB with the addition of radical initiator. Reaction was performed with the following conditions, [PBzMA-DTB]:[ABCN]= 1:0.2, Solvent= TCB, T= 120 °C, $[RU]_0$= 5 mM.

Table S2: Depropagation rate constants, k_{dp}^{app}, of the depolymerization reactions of PBzMA-DTB with (0.2 equiv. ABCN) and without the addition of radical initiator ABCN at 120 °C. The constants were calculated directly from the gradients of -ln(1-ρ) vs time plots (Figure 1b).

	k_{dp}^{app} (s^{-1})
**No initiator**	4.5 x 10^{-5}
**0.2 eq ABCN**	0.0021
**0.5 eq ABCN**	0.0032
**1 eq ABCN**	0.0032

Table S3: Effect of different amounts of initiator on the depolymerization of PBzMA-DTB. Reaction conditions: $[RU]_0$= 5 mM, Solvent= TCB, V_{tot}= 6 mL, T= 120 °C. Samples were taken after 3, 5, 10, 15, 30 and 60 minutes.

Time (mins)	Depol. Conversion %				
	Entry 1 0.2 eq	Entry 2 0.5 eq	Entry 3 1 eq	Entry 4 2 eq	Entry 5 5 eq
**3**	22	28	42	53	62
**5**	43	56	74	77	72
**10**	73	85	85	79	73
**15**	83	86	85	79	73
**30**	87	87	85	80	68
**60**	87	87	85	80	68

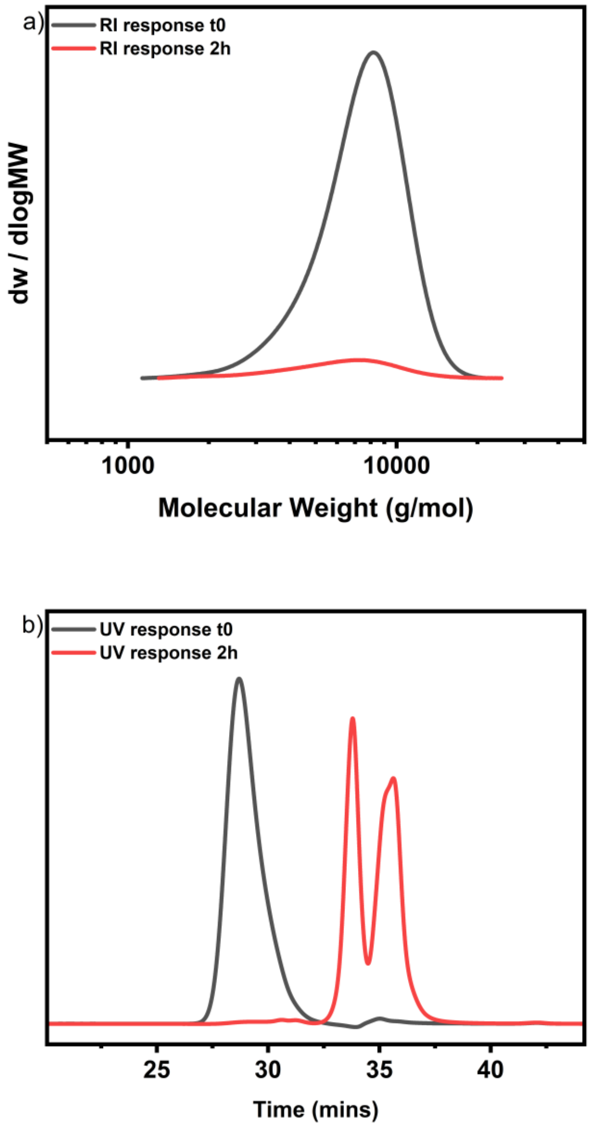

Figure S5: a) RI and b) UV response of PBzMA-DTB polymer before (t0, black) and after 2h (red) of the depolymerization reaction in the presence of 0.2 equiv. of radical initiator (ABCN). Depolymerization conversion was calculated to be 87%. The UV intensity signal decreased more than 99% after 2h. Reaction conditions were: Solvent=TCB, T=120 °C, $[RU]_0$= 5 mM. For the preparation of the SEC samples ~ 2 mL of the solution was blow dried, dissolved in 1.3 mL of DMAc and passed through a syringe filter (0.45 μM PTFE membrane).

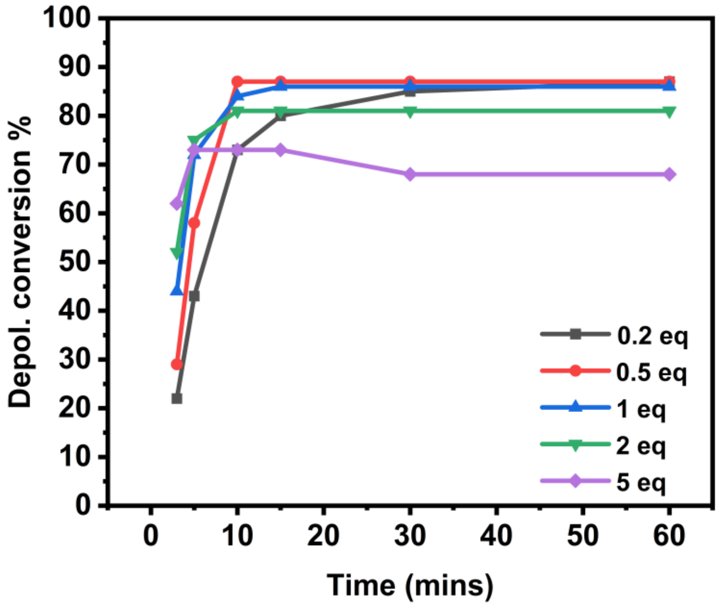

Figure S6: Effect of different amounts of initiator on the rate and final conversion of the depolymerization of PBzMA-DTB. Reaction conditions: $[RU]_0$= 5 mM, Solvent= TCB, V_{tot}= 6 mL, T= 120 °C.

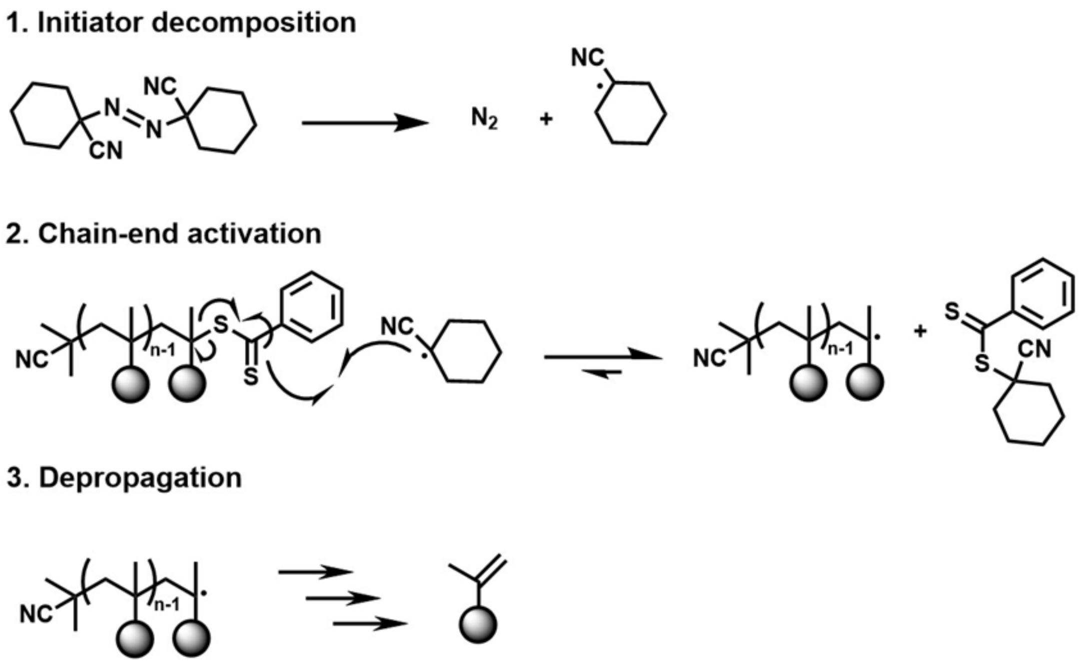

Scheme S4: The pathway for the depolymerization of polymethacrylates in the presence of a radical initiator.

Table S4: RAFT Depolymerization of PBzMA in different solvents with the addition of radical initiator. The samples were taken after 2 hours. Reaction conditions: [PBzMA-DTB]:[ABCN]= 1:0.2 , $[RU]_0$= 5 mM, V_{tot}= 6 mL, T= 120 °C.

Solvent	Depol. Conversion %
Toluene	88
TCB	87
DMSO	92
MeCN	89
DCB	87
Xylene	80
Anisole	85

Table S5: RAFT depolymerization of PBzMA-DTB in different solvents in the absence of radical initiator. The samples were taken after 2 hours. Reaction conditions: $[RU]_0$= 5 mM, V_{tot}= 6 mL, T= 120 °C.

Solvent	Depol. Conversion %
Toluene	41
TCB	23
DMSO	59
MeCN	56
DCB	9
Xylene	66
Anisole	31

Table S6: RAFT depolymerization of PBzMA-DTB in dioxane in the presence and absence of radical initiator. Reaction conditions: [RU]0= 5 mM, Vtot= 6 mL, T= 120 °C.

	**Depol. Conversion %**	
**Time (mins)**	**0 eq ABCN**	***0.2 eq ABCN***
**5**	-	41
**10**	-	73
**15**	20	86
**30**	45	90
**60**	63	90
**120**	86	90
**240**	89	90
**480**	90	90

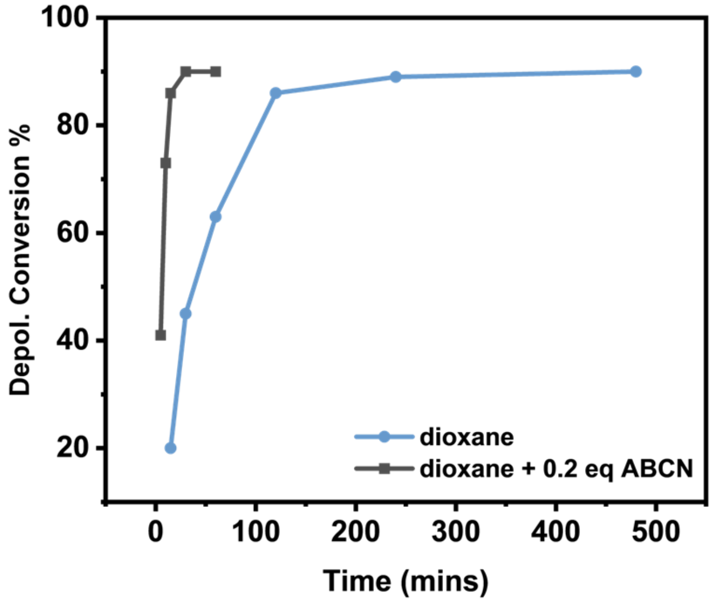

Figure S7: RAFT depolymerization of PBzMA-DTB in dioxane in the presence and absence of radical initiator. Reaction conditions: $[RU]_0$= 5 mM, V_{tot}= 6 mL, T= 120 °C.

Table S7: Effect of higher temperatures on the depolymerization of PBzMA-DTB with the addition of radical initiator. The depolymerization reactions were conducted at 140 °C and 170 °C in TCB. ([PBzMA-DTB]:[ABCN]= 1:0.2, $[RU]_0$= 5 mM, V_{tot}= 6 mL).

**Time (mins)**	**Depol. Conversion %**	
	**Entry 1 140 °C**	**Entry 2 170 °C**
**1**	4	17
**2**	22	78
**3**	51	83
**5**	75	86
**10**	86	86
**15**	86	86
**30**	86	86
**60**	86	86

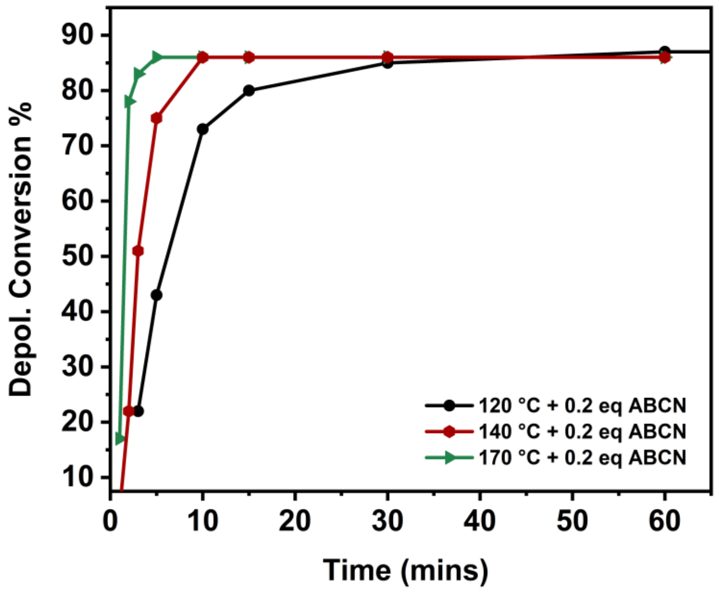

Figure S8: Kinetics of the depolymerization of PBzMA-DTB with radical initiator at 120 °C, 140 °C and 170 °C. The reaction conditions were: [PBzMA-DTB]:[ABCN]= 1:0.2, $[RU]_0$= *5 mM*, Solvent= TCB, V_{tot}= 6 mL.

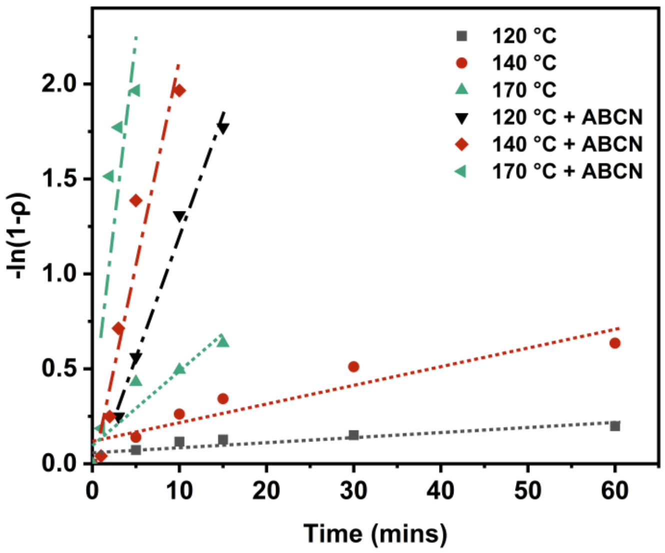

Figure S9: Kinetic plots of the depolymerization of PBzMA-DTB in TCB at different temperatures with and without initiator. The repeat unit concentration was in each case 5 mM and ρ= the decimalized depolymerization conversion. The k_{dp}^{app} for the reactions were 4.5 x 10^{-5}, 2.7 x 10^{-4} and 0.0010 s^{-1} without initiator and 0.0021, 0.0049, 0.011 s^{-1} with initiator at 120 °C, 140 °C and 170°C, respectively.

Table S8: Depropagation rate constants, k_{dp}^{app}, of the depolymerization reactions of PBzMA-DTB with (0.2 equiv. ABCN) and without the addition of radical initiator ABCN at 140 °C. The constants were calculated directly from the gradients of -ln(1-ρ) vs time plots (Figure S8).

	k_{dp} ^{app} (s^{-1})
**No initiator**	2.7 x 10^{-4}
**0.2 eq ABCN**	0.0049

Table S9: Depropagation rate constants, k_{dp}^{app}, of the depolymerization reactions of PBzMA-DTB with (0.2 equiv. ABCN) and without the addition of radical initiator ABCN at 170 °C. The constants were calculated directly from the gradients of -ln(1-ρ) vs time plots (Figure S8).

	k_{dp} ^{app} (s^{-1})
**No initiator**	0.0010
**0.2 eq ABCN**	0.011

Table S10: Thermal RAFT depolymerization of PBzMA-DTB in TCB at 140 °C and 170 °C without radical initiator. Reaction conditions: $[RU]_0$= 5 mM, Solvent= TCB, V_{tot}= 6 mL.

**Time (mins)**	**Depol. Conversion %**	
	**Entry 1 140 °C**	**Entry 2 170 °C**
**5**	13	35
**10**	23	46
**15**	29	47
**30**	40	48
**60**	47	48
**120**	50	48

Table S11: Depolymerization of PBzMA-DTB at 100 °C with different amounts of ABCN radical initiator. Reaction conditions: $[RU]_0$= 5 mM, Solvent= TCB, V_{tot}= 6 mL.

Time (mins)	Conversion %			
	Entry 1	Entry 2	Entry 3	Entry 2
	0 eq ABCN	0.2 eq ABCN	1 eq ABCN	2 eq ABCN
**15**	0	8	35	41
**30**	0	23	57	64
**60**	0	52	76	80
**120**	0	68	80	80
**180**	0	81	81	80

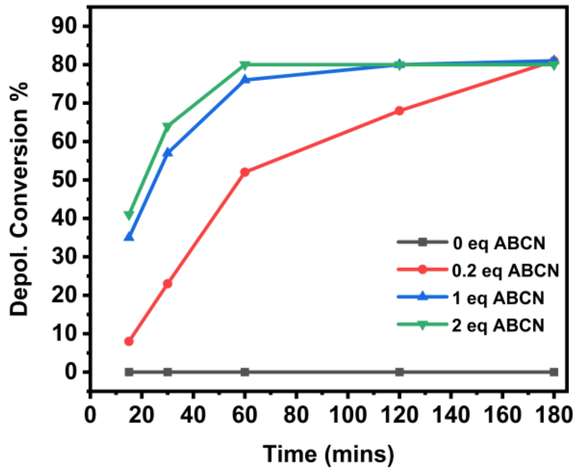

Figure S10: Effect of different initiator concentrations on the kinetics and the final conversions of the PBzMA-DTB depolymerization reaction at 100 °C. The reaction conditions were: $[RU]_0$= 5 mM, Solvent= TCB, V_{tot}= 6 mL.

Table S12: Depolymerization of PBzMA-DTB at 80 °C using ABCN or AIBN as the radical initiator. $[RU]_0$= 5 mM, Solvent= TCB, V_{tot}= 6 mL. The samples were taken after 3 h of reaction time.

Initiator	Depol. Conversion %
**0.2 eq ABCN**	10
**0.2 eq AIBN**	15
**1 eq AIBN**	58

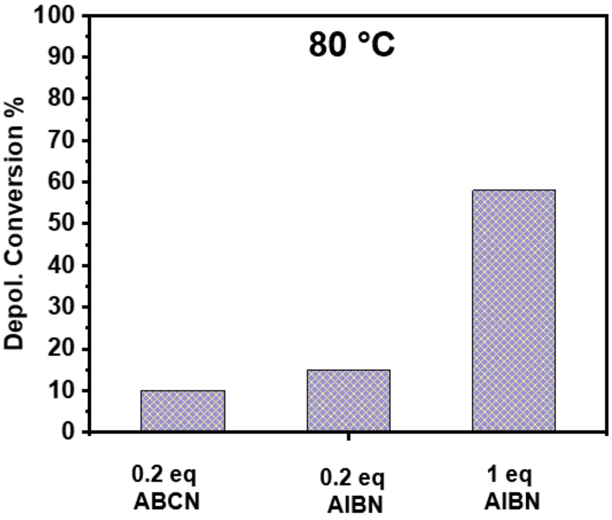

Figure S11: Bar graph showing the conversions after 3 h of depolymerization of PBzMA-DTB at 80 °C. Reaction conditions: $[RU]_0$= 5 mM, Solvent= TCB, V_{tot}= 6 mL.

Table S13: Higher concentration depolymerization of PBzMA-DTB performed at 120°C with and without radical initiator. The depolymerization conversions were measured after 2 hours of reaction time. The reactions were performed at concentrations ranging from 5 -500 mM $[RU]_0$. Reaction conditions: [PBzMA-DTB]:[ABCN]= 1:0.2, Solvent= TCB, V_{tot}= 3 mL.

**[RU] mM**	**Depol. Conversion %**	
	**Entry 1** **No Initiator**	**Entry 2** **0.2 eq ABCN**
**5**	23	87
**10**	17	83
**25**	15	82
**50**	14	78
**100**	12	72
**250**	7	50
**500**	4	35

Table S14: Higher concentration depolymerization of PBzMA-DTB performed at 140°C with and without radical initiator. The depolymerization conversions were measured after 2 hours of reaction time. The reactions were performed at concentrations ranging from 5 - 500 mM $[RU]_0$. Reaction conditions: [PBzMA-DTB]:[ABCN]= 1:0.2, Solvent= TCB, V_{tot}= 3 mL.

**[RU] mM**	**Depol. Conversion %**	
	**Entry 1** **No Initiator**	**Entry 2** **0.2 eq ABCN**
**5**	50	87
**10**	49	87
**25**	36	84
**50**	35	84
**100**	18	83
**250**	13	68
**500**	11	54

Table S15: Higher concentration depolymerization of PBzMA-DTB performed at 170°C with and without radical initiator. The depolymerization conversions were measured after 2 hours of reaction time. The reactions were performed at concentrations ranging from 5 - 500 mM $[RU]_0$. Reaction conditions: [PBzMA-DTB]:[ABCN]= 1:0.2, Solvent= TCB, V_{tot}= 3 mL.

.

[RU] mM	Depol. Conversion %	
	Entry 1 No Initiator	Entry 2 0.2 eq ABCN
**5**	48	87
**10**	54	87
**25**	51	85
**50**	47	84
**100**	41	82
**250**	30	78
**500**	23	61

References

(1) Perrier, S. 50th Anniversary Perspective: RAFT Polymerization—A User Guide. *Macromolecules* **2017**, *50* (19), 7433-7447. DOI: 10.1021/acs.macromol.7b00767.
